# In Memory of the Late Dr. W. H. Dwindle

**Published:** 1896-06

**Authors:** 


### In Memory of the Late Dr. W. H. Dwinelle.

We understand that a biographical and historical sketch of the late Dr. W. H. Dwinelle is being prepared by his sister, Miss Louisa L. Dwinelle. This will be not only a loving tribute from a dear sister, but will undoubtedly present matter of great interest to the profession at large concerning Dr. Dwinelle's career, which was a very remarkable one--remarkable for the length of his professional life, and especially remarkable for the work he did for his beloved profession during that period. He was practically a pioneer, was familiar with pioneer methods and processes, many of which he found crude and which under his master hand were changed into more feasible and practicable forms and conditions. He devoted much time and labor to this kind of work, and the history of what he did and how he did it would be of lasting benefit, especially to the younger members of the profession. We shall look with interest to the forthcoming of this work.

